# The Progress on Magnetic Material Thin Films Prepared Using Polymer-Assisted Deposition

**DOI:** 10.3390/molecules28135004

**Published:** 2023-06-26

**Authors:** Hongtao Ren, Jing Zhong, Gang Xiang

**Affiliations:** 1 School of Materials Science and Engineering, Liaocheng University, Liaocheng 252000, China; 2 College of Physics, Sichuan University, Chengdu 610064, China; 15982352608@163.com

**Keywords:** polymer-assisted deposition (PAD), ZnO, NiO, MoS_2_, MoSe_2_, ReS_2_

## Abstract

Polymer-assisted deposition (PAD) has been widely used in the preparation of high-quality oxides and sulfides for basic research and applications. Specifically, diverse PAD-prepared magnetic material thin films such as ZnO, Ga_2_O_3_, SrRuO_3_, LaCoO_3_, LaMnO_3_, Y_3_Fe_5_O_12_, MoS_2_, MoSe_2_, and ReS_2_ thin films have been grown, in which thickness-dependent, strain-modulated, doping-mediated, and/or morphology-dependent room-temperature ferromagnetism (RTFM) have been explored. Inspired by the discovery of intrinsic low-temperature FM in two-dimensional (2D) systems prepared using mechanical exfoliation, the search for more convenient methods to prepare 2D ferromagnetic materials with high-temperature FM has seen explosive growth, but with little success. Fortunately, the very recent synthesis of 2D NiO by PAD has shed light on this challenge. Based on these abovementioned developments, the difficulties of PAD when preparing a-few-nanometer single-crystalline materials and the opportunities in PAD for novel materials such as chiral magnetic soliton material Cr_1/3_NbS_2_ are discussed.

## 1. Introduction

Since polymer-assisted deposition (PAD) was proposed by Jia et al. to prepare metal oxides in 2004 [1], it has been widely used in the preparation of various materials in many fields, including spintronics [2,3,4], energy storage and conversion [5,6], nuclear engineering [7,8,9], conformal coating [10,11,12], superconductivity [13,14,15], and optoelectronics [16,17,18,19]. PAD is a chemical synthesis method in which metal ions coordinated with polymers are used as the precursor, and then the polymer is removed at a high temperature (approximately 500 °C) [1,2,3,4,5,20,21,22,23,24]. Owing to the unique properties of its polymer-involved process, PAD has five main advantages: (1) it can realize wafer-scale preparation of materials [2,3,4,5,19,20,25]; (2) it can accurately adjust the thickness in the range of sub-nanometer thickness to a thickness of hundreds of nanometers [2,5,17,26,27,28]; (3) it is easy to produce materials doped with a variety of metal elements [29,30,31,32,33,34]; (4) it can prepare materials with different morphologies, including thin films (TFs) [1,35,36], composites [32,37,38], epitaxial heterostructures [19,28,31,39,40,41,42], nanowires (NWs) [43,44], hybrid fibers [11], feathers-like nanostructures [45,46], and superlattices [1,47]; and (5) it can prepare metal oxides [8,30,31,34,48,49,50,51,52,53,54], metal nitrides [13,15,55,56,57], metal carbides [14,58], single-element materials [12,16,59], transition metal chalcogenides (TMDs) [3,4,5,19,25,26,60], and other types of materials [18]. These advantages make it possible to reveal thickness-dependent [27,61,62,63,64,65], strain-mediated [5,22,27,63,65,66,67,68,69], doping-modulated [29,70], and morphology-dependent [21,29,43,44,64,71] RTFM in the PAD-prepared materials.

**Figure 1:**
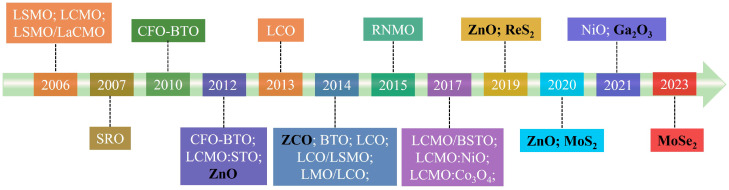
Timeline showing key developments of ferromagnetic materials prepared using PAD [2,3,4,27,29,30,31,33,35,37,40,41,43,44,45,46,50,52,63,67,71,76,77]. White font represents the experimental progress; black bold font represents the progress by our group. LSMO [30]: La_0.67_Sr_0.33_MnO_3_; LCMO [30]: La_0.67_Ca_0.33_MnO_3_; LSMO/LCMO [31]: La_0.67_Sr_0.33_MnO_3_/La_0.67_Ca_0.33_MnO_3_; SRO [33]: SrRuO_3_; CFO-BTO [45,76]: CoFe_2_O_4_–BaTiO_3_; LCMO: STO [37]: La_0.67_Ca_0.33_MnO_3_: SrTiO_3_; LCO [27,63,67]: LaCoO_3_; ZCO [29]: Zn_1−x_Co_x_O; LMO [40,41]: LaMnO_3_; RNMO [77]: Re_2_NiMnO_6_ (Re = La, Pr, Nd, Sm, Y); LCMO/BSTO [52]: La_0.8_Ca_0.2_MnO_3_/Ba_0.8_Sr_0.2_TiO_3_. YIG [78]: Y_3_Fe_5_O_12_.

In this review, we first summarize the developments of PAD-grown ferromagnetic materials. The typical magnetic materials prepared using PAD are shown in chronological order in Figure 1. The structural and magnetic properties, as well as the modulation of RTFM in these materials, are presented and discussed. Then the challenges and opportunities for PAD in the preparation of 2D ferromagnetic materials are also manifested. In the end, we compare the advantages of PAD, mechanical exfoliation (ME) [72], and chemical vapor transport [73,74,75] (CVT) in the preparation of novel materials such as chiral magnetic soliton material CrNbS_1/3_ (CNS).

## 2. Polymer-Assisted Deposition (PAD)

### 2.1. Main Processing Steps

**Figure 2:**
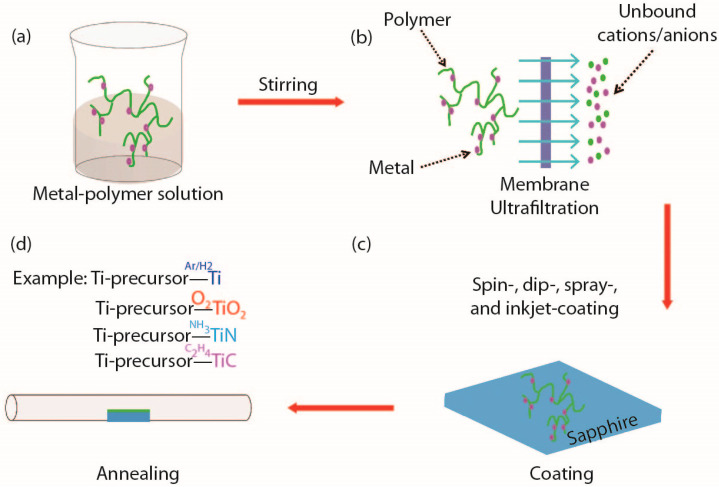
(**a**–**d**) Schematic illustration of the main processing steps used to grow thin films using PAD [20].

Similar to other traditional chemical solution deposition (CSD) methods, PAD also involves preparing precursor solutions using coatings and heat treatment of precursor films. As shown in Figure 2, the main difference lies in the preparation of polymer precursor solutions (Figure 2a) and ultrafiltration (Figure 2b). In PAD, metal ions coordinate with polymers in the solution to form precursors. This polymer mainly refers to polyethyleneimine (PEI), which can complex with the vast majority of metals. 

More importantly, the polymer has three unique characteristics [20,53,54], as follows: (1) universality. For the first-row transition metal elements, simple PEI can coordinate with them to form covalent complexes. For other hard-to-bind metals, functionalized PEI with carboxylic acids can be used to achieve coordination with them. Moreover, the protonation PEI can also coordinate with anionic metal complexes. In detail, EDTA can form stable complexes with almost all metals, and then the complexes successfully bind to PEI. (2) Stability. The polymer solution can be stored in air for several months, and its viscosity can be adjusted by simply removing water or diluting with deionized water under vacuum conditions. Polymer precursors with different metal elements can be mixed in any ratio to achieve easy doping. (3) Conformality. Spin-coating can be achieved not only on flat substrates, but also on curved substrates such as carbon nanotubes fibers [11], quartz fiber [12]*,* and porous materials [10] (Figure 2c). In addition, using the same Ti-polymer precursor solution, different Ti-based nanomaterials can be obtained under different atmospheres, such as Ti, TiO_2_ [1,79], TiN [55,56], and TiC [11,58]. 

### 2.2. Advantages

Compared to other CSD methods, PAD has four obvious advantages: (1) it can realize low-cost growth of wafer-scale materials [2,3,4,5,19,20,60]; (2) it can accurately adjust the thickness [2,5,17,26,27,28] with the precursor concentration and spin-coating rates [2,26]; (3) it can prepare nanostructures with various morphologies, including TFs [1,35,36], composites [32,37,38], epitaxial heterostructures [19,28,31,39,40,41,42], NWs [43,44], hybrid fibers [11], feathers-like nanostructures [45,46], multilayered structures [47], and superlattices [1,47]; and (4) it can prepare single and complex metal oxides [8,30,31,34,48,49,50,51,52,53,54], metal nitrides [13,15,55,56,57], metal carbides [14,58], single-element materials [12,16,59], TMDs [3,4,5,19,25,26,60], and other types of materials [18].

## 3. Traditional Oxide-Based Magnetic Semiconductor Thin Films

### 3.1. ZnO

Since Dietl et al. [80] predicted that the Curie temperature (*T_c_*) of Co-doped ZnO could exceed 300 K through the Zener model, researchers focused primarily on the ferromagnetism of transition-metal (TM) doped ZnO [81,82]. Remarkably, undoped ZnO can also show RTFM [83,84]. Undoped ZnO nanostructures with RTFM were experimentally obtained through various methods, including sol-gel [84,85,86], pulsed laser deposition (PLD) [87,88], ball milling (BM) [89,90], PAD [29,43,44,71], electrochemical deposition method [91], chemical vapor deposition (CVD) [92], and ionic layer epitaxy (ILE) [64]. In general, the RTFM in ZnO is dependent on its morphology [21,43,44,71]. 

In 2006, Lin and Xie et al. [93] prepared ZnO TFs on sapphire substrates by means of PAD. The films were composed of nanometric particles and had a high c-axis orientation. The crystallinity, c-axis orientation, and surface morphology of ZnO TFs could be modified with rapid heat treatment. It was found that more oxygen vacancies (*Vo*) could enhance the green photoluminescence properties. 

In 2012, we developed ZnO TFs using PAD [71] where all the films exhibited RTFM. Results from the superconducting quantum interference device (SQUID) and X-ray photoelectron spectroscopy (XPS) show that RTFM was not directly related to *Vo*. In order to clarify the origin of RTFM, we tested the samples using positron annihilation spectroscopy (PAS). Unexpectedly, zinc vacancies (*V_Zn_*) were found to be responsible for the RTFM. Actually, this phenomenon of RTFM caused by *V_Zn_* was also found in samples prepared using PLD [87,94] and ILE [64]. In contrast, it was reported that *Vo* was the origin of RTFM in the undoped ZnO samples’ prepared sol-gel [84] and pulsed electron beam deposition (PED) [95].

**Figure 3:**
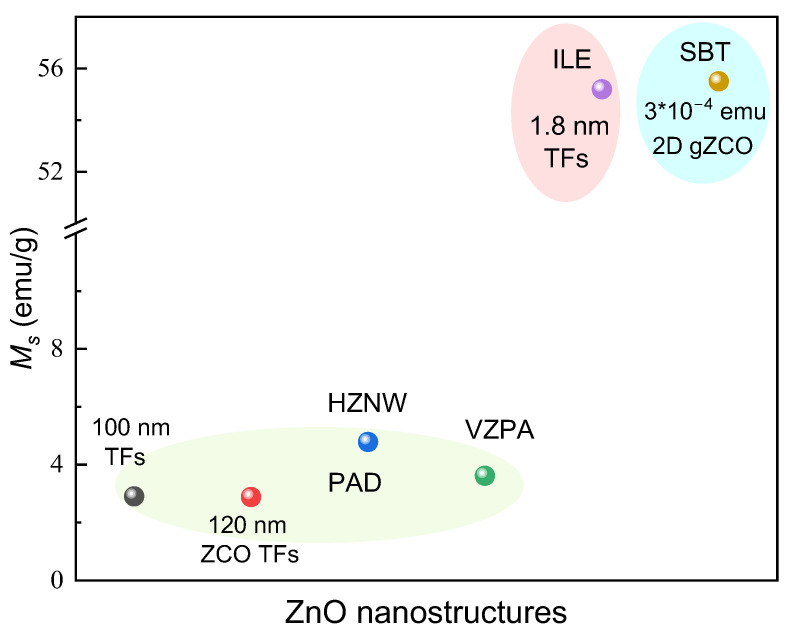
*M_s_* of various ZnO nanostructures. ZCO: Zn_1−x_Co_x_O; gZCO: graphitic Zn_1−x_Co_x_O. HZNW: horizontal ZnO nanowire arrays. VZPA: vertical ZnO nanopillar arrays. SBT: a solution-based and template-assisted method. Data from [29,43,44,64,70,71].

A variety of ferromagnetic ZnO nanostructures, including ZnO TFs, Zn_0.97_Co_0.03_O TFs, horizontal ZnO nanowire arrays (HZNW) [43], and vertical ZnO nanopillar arrays (VZPA) [44], have been prepared using PAD. As shown in Figure 3, the RTFM of ZnO nanostructures with different morphologies is obviously different.

### 3.2. Ga_2_O_3_

High-quality β-Ga_2_O_3_ TFs [96] with a single preferential growth orientation of (−201) and low defect density were successfully grown on sapphire substrates using PAD. Interestingly, the Mn concentration was closely related with the saturation magnetization (*M_s_*) and coercive field (*H_c_*) values in amorphous Mn-doped gallium oxide [97] TFs, which are different from crystalline Mn-doped Ga_2_O_3_ TFs. Notably, the samples annealed in air exhibited a *M_s_* [98] as strong as 170% times that of the samples annealed in pure O_2_ gas, which can be quantitatively explained by oxygen-vacancy-controlled ferromagnetism due to bound magnetic polarons established between delocalized hydrogenic electrons of *Vo*s and local magnetic moments of Mn^2+^, Mn^3+^, and Mn^4+^ ions in the samples.

### 3.3. SrRuO_3_ (SRO)

**Figure 4:**
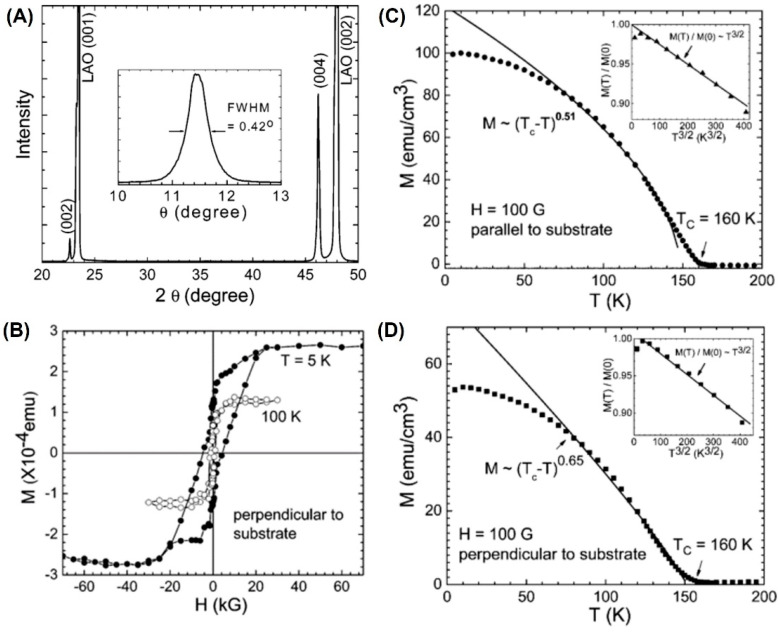
Thickness-dependent magnetism in SRO. (**A**) Typical θ-2θ XRD spectrum of an SRO film grown on an LAO substrate annealed at 550 °C. The inset shows the ω-rocking curve of the (002) SRO reflection. (**B**) Magnetization versus magnetic field (M-H) hysteresis loops with the magnetic field perpendicular to the substrate surface at 5 and 100 K. Temperature dependence of field-cooled magnetization of an SRO film annealed at 550 °C, where the field is (**C**) parallel and (**D**) perpendicular to the substrate surface (reprinted with permission from [33]).

SrRuO_3_, as a ferromagnetic conductive material, is usually used as a metal layer at magnetic tunnel junctions [99]. Its unique perovskite structure facilitates integration with other functional metal oxides. Epitaxial ferromagnetic SRO TFs were obtained experimentally through several experimental methods, including PLD [99,100,101], sol-gel [102,103], sputtering [104,105,106], molecular beam epitaxy (MBE) [107], and metalorganic chemical vapor deposition (MOCVD) [108,109]. PAD has its unique advantages as a low-cost method for preparing large-area metal oxides coatings. In 2007, Jia et al. [33] successfully prepared epitaxial SRO TFs with high crystallinity using PAD, as shown in Figure 4A. Their curie temperature (*T_c_*) is about 160 K in Figure 4B–D, which is equivalent to that in the other literature [104]. The thickness of PAD-prepared SRO TFs is usually around 100 nm.

### 3.4. Perovskite

#### 3.4.1. LaCoO_3_ (LCO)

**Figure 5:**
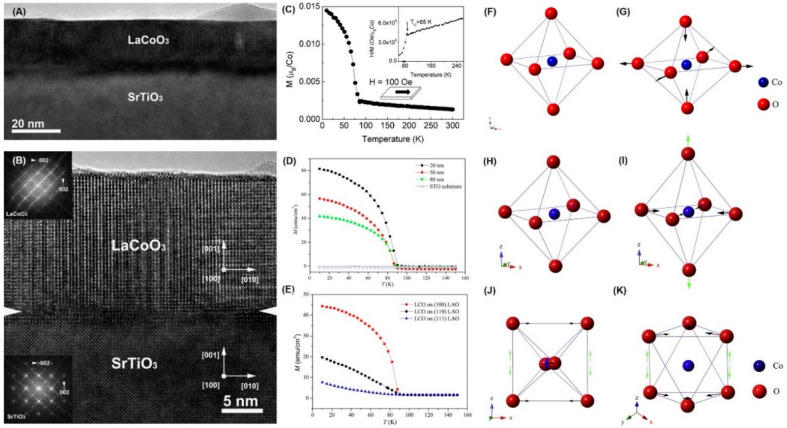
Thickness-dependent and strain-induced FM in LCO. (**A**) Cross-section TEM image of an LCO film on STO. (**B**) High-resolution TEM and electron diffraction patterns showing the epitaxial growth of the film on the substrate. (**C**) Magnetization curves of the LCO film. The inset shows the temperature dependence of the inverse susceptibility (reprinted with permission from [69]). (**D**) M-T curves of the LCO epitaxial thin films with different thicknesses (reprinted with permission from [65]). (**E**) M-T curves of the 60 nm thick (100), (110), and (111) oriented LCO epitaxial TFs on LAlO_3_ (LAO) substrates (reprinted with permission from [27]). Schematic diagram of biaxial tensile strain-induced distortion of CoO_6_ octahedron: (**F**,**H**) the regular CoO_6_ octahedron in bulk LCO, (**G**) the compressed CoO_6_ octahedron along the c axis due to the biaxial tensile strain in the ab plane, biaxial compressive strain-induced distortion of CoO_6_ octahedron for (**I**) (100), (**J**) (110), and (**K**) (111) oriented LCO films grown on (100), (110), and (111) LAO substrates, respectively (reprinted with permission from [65]; reprinted with permission from [27]).

Goodenough et al. [110] discovered the phase transition of Co from the low-spin state to the high-spin state in LCO samples, which were grown using high-temperature ceramic techniques. However, it was very difficult to control the sample uniformity and oxygen dose. Later, they obtained uniform single-phase LCO samples using precursor coprecipitation [111] and studied the magnetic and transport properties. The temperature-dependent electronic structure model can explain this phase transition from low-spin Co (III) to high-spin Co^3+^ well. In 2013, Rivadulla et al. [67] successfully prepared high-quality epitaxial LCO TFs on SrTiO_3_ (STO) using PAD. The film was uniform and smooth throughout the region, as shown in Figure 5A. As shown in Figure 5B, LCO films were grown epitaxially on STO substrates. Its *T_c_* is around 85 K in Figure 5C. The thickness-dependent magnetism [27,63] in Figure 5D has also been found in LCO film systems, which is closely related to biaxial tensile strain. When the film thickness is reduced, the FM is enhanced.

LCO TFs induced an additional thermal strain through thermal expansion coefficient mismatch [65], which would lead to larger lattice parameters. Interestingly, *T_c_* and magnetism can be significantly increased by adjusting the in-plane tensile strain. However, for PAD-grown epitaxial LCO TFs, in-plane lattice tensile strain and CoO_6_ octahedral rotation occur. In the epitaxial growth process, in-plane biaxial tensile deformation is induced, causing the Co-O distance (r_Co−O_) to stretch in the in-plane direction and compress in the out-of-plane direction, as shown in Figure 5F–G. The inhibition of the CoO_6_ octahedral rotation is more evident in thinner films. In addition, the rectangular distortion of the CoO_6_ octahedron caused by the deformation can also reduce the e_g_-t_2g_ gap [63]. In general, the strain-induced FM originates from the reduction in the e_g_-t_2g_ gap and the inhibition of the CoO_6_ octahedral rotation. 

Notably, the orientation of LAO substrates has a significant effect on the epitaxial film magnetism, as in Figure 5E. The LCO films with three different orientations show significantly different magnetic behaviors from the bulk, which is related to the biaxial compressive strain and the tetragonal distortion of CoO_6_ octahedra (Figure 5H–K). It was also found that PEI molecular weight, heat treatment conditions, and spin-coating rate had significant effects on the crystallinity and epitaxial quality of LCO TFs [28]. In addition, epitaxial LCO TFs were obtained with PLD [69]. Similarly, the FM to PM phase transition was also observed at about 80 K.

**Figure 6:**
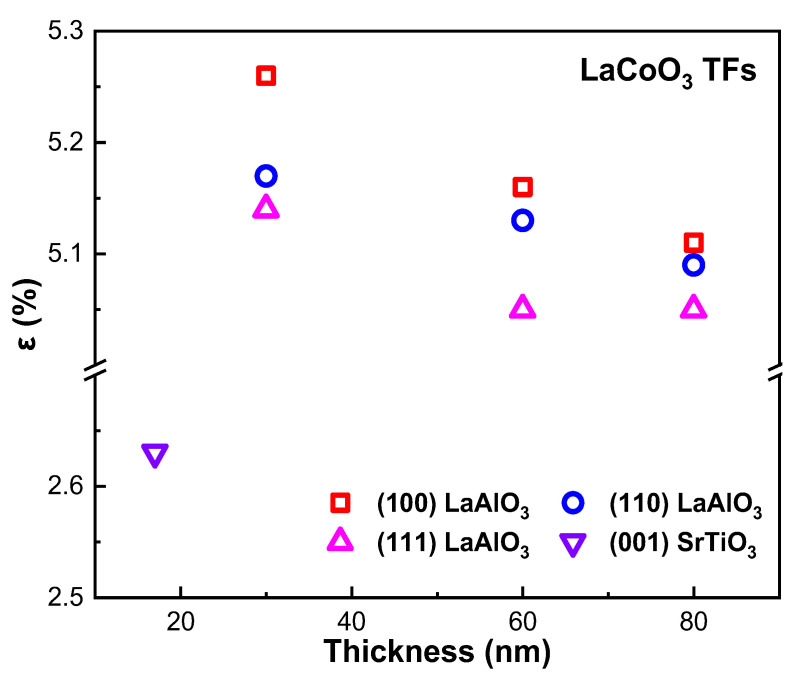
Strain difference in LCO TFs [27,69] with different thicknesses caused by different crystal orientations of LAO and STO substrates.

Modulating material properties through strain [22,112,113,114] has always been a mysterious topic, especially the regulation of RTFM [4,115,116,117]. However, it has always been a challenge to introduce strain directly into materials in experiments. Although strain has been introduced into materials with many methods, such as using flexible substrates [118], using lattice mismatch [118,119] or thermal [120,121] strain, introducing wrinkles, and alloying [122], these methods require additional equipment [123,124,125]. Tensile strain [27,67] is introduced into LCO TFs by using LaAlO_3_ substrates with different lattice orientations in Figure 6. 

#### 3.4.2. LaMnO_3_ (LMO)

In 1965, Solovyev et al. [126] explained the AFM of LMO perovskite by using the itinerant-electron model based on the local spin density approximation. Lattice distortion strongly affects magnetism. When La^3+^ is partially placed, the FM is introduced into the LMO system. As a typical example, Rivadulla et al. [127] reported an oriented LMO with RTFM grown using PAD, which is up to a number of centimeters in size and is greatly compatible with standard microfabrication techniques. Furthermore, they found that the type of substrate affects the orientation of LMO films [128], and some non-stoichiometric diffraction peaks may even appear. Even after annealing at 900 °C in air, the epitaxial characteristics of LMO TFs were still preserved. 

Jain et al. [30] attempted to prepare high-quality Sr- and Ca-doped LMO epitaxial films using PAD. The annealing temperature determined the crystallinity, microstructure, resistance, magnetism, and magnetoresistance of TFs. In addition, dense polycrystalline films of La_1−x_Ca_x_MnO_3_ [129] were also deposited on Si substrates. Moreover, it was found that the annealing atmosphere could affect the morphology of TFs, and the film was composed of sintered grains after annealing under oxygen. Similarly, the *T_c_* and *M_s_* could also be modulated through annealing conditions. 

Generally, the introduction of the second phase will also greatly modulate the properties of the materials. SrTiO_3_ [50], as the second phase, was introduced to regulate the magnetic transport properties of LCMO epitaxial TFs using PAD. This phase increases the resistance of LCMO TFs by increasing the height of the spin-dependent tunneling barrier between magneto-crystals and significantly changes the magnetoresistance (MR) by increasing the MR value and reducing the metal–insulator transition temperature. Similarly, NiO or Co_3_O_4_ are also added, which can have a similar effect [37]. Anti-ferromagnetic Co_3_O_4_ has a more obvious inhibitory effect on the magnetism of LCMO TFs.

PAD-grown epitaxial multilayer TFs were also systematically studied. The behavior of the interfacial magnetic coupling of LCO/LMO [41] systems was studied by Rivadulla et al. The LCO precursor is spin-coated after the preparation of LMO TFs, and the LCO/LMO epitaxial bilayers can be prepared after annealing. The coercivity of LCO/LMO is 30 times higher than that of single-film LMO or LCO due to superexchange related to a redox reaction at the interface. However, the coercivity of La_0.92_MnO_3_/LCO [42] is 5 times higher than that of LMO film, which is caused by FM superexchange at the interface. Furthermore, the tunneling conduction phenomenon was also found in ferromagnetic epitaxial bilayers of LCO/LSMO [40]. The negative temperature coefficient of tunneling resistance shows that the quality of the heterojunction meets the basic research and application. Interestingly, substrate orientation also has a significant effect on the magnetic and transport properties of the LCMO/BSTO bilayer films [52]. In addition, metal–insulator phase transition highly related to substrate orientation was also found. Moreover, a large magnetoresistance [31] (−71% at 5T) was observed in multilayer-coated LSMO/LCMO TFs. Therefore, spin-coating multilayer ferromagnetic materials is an effective way to modulate phase transition temperature and magnetoresistance.

**Figure 7:**
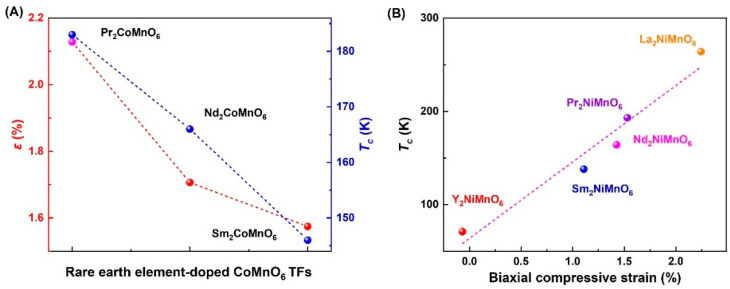
(**A**) Biaxial compressive strain induced by doping rare earth elements and *T_c_* in RCMO TFs [68]. (**B**) The evolution of *T_c_* and biaxial compressive strain in RNMO TFs [77].

Easy doping [1,29,30,31,32,34,36,51,130] is an important feature of PAD. Sr-doped LaMnO_3_ [30,31,40,50,52] TFs and Co-doped ZnO [29] TFs are representatives of magnetic materials prepared using PAD. Doping can not only mediate the magnetic strength [29], but also drive the magnetic phase transition [30,31]. Rare earth metal element doping can introduce the biaxial compressive strain in RCMO [68] and RNMO [77] TFs, as seen in Figure 7A,B. Due to the ionic radius difference of rare elements, the in-plane lattice parameter is changed, and the biaxial compressive strain is introduced in the film due to the lattice relaxation effect. Moreover, there is a linear relationship between *T_c_* and biaxial compressive strain. Furthermore, the preparation of a monolayer-doped system using PAD is a challenging research direction.

#### 3.4.3. Y_3_Fe_5_O_12_ (YIG)

**Figure 8:**
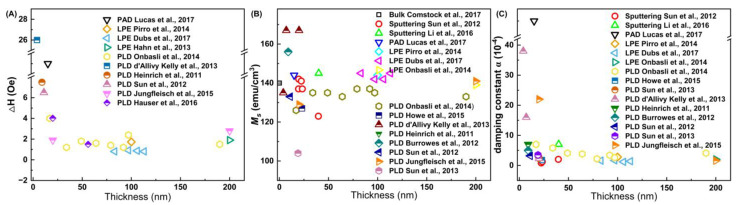
Comparison of magnetic data for YIG TFs [78,131,133,137,139,140,141,142,143,144,146,147,148,149,152]. (**A**) Room-temperature peak-to-peak linewidth (ΔH). (**B**) Saturation magnetization (*M_s_*). (**C**) The Gilbert damping parameter (α).

Yttrium iron garnet (Y_3_Fe_5_O_12_: YIG) single crystal material [131,132,133] is a kind of ferromagnetic insulator which has been widely used in spintronics [78], magnonics [134], and spin caloritronics [135,136] in recent years. However, most experimental methods, such as liquid phase epitaxy (LPE) [131,137,138,139], pulsed laser deposition (PLD) [140,141,142,143,144,145,146,147,148,149], and sputtering [150,151,152], have difficulties controlling the thickness of TFs within the nanometer range. Notably, epitaxial YIG TFs with a thickness of about 15 nm were obtained using PAD [78]. Epitaxial TFs have ferromagnetic resonance properties comparable to other methods in Figure 8 and can be applied to spintronics and high-frequency applications.

## 4. Layered Material Thin Films

Since long-range intrinsic ferromagnetic order was observed in exfoliated Cr_2_Ge_2_Te_6_ [153] and CrI_3_ [62] monolayer systems using polar magneto-optical Kerr effect (MOKE) measurements in 2017, the research on 2D ferromagnetic materials has become a fast-growing field. However, the size and thickness of 2D materials obtained with most of the methods, including mechanical exfoliation [62,153,154], chemical vapor transport [63,155,156,157], microwave irradiation [158], direct vapor transport technique [159], and so on, are difficult to control.

### 4.1. MoS_2_

**Figure 9:**
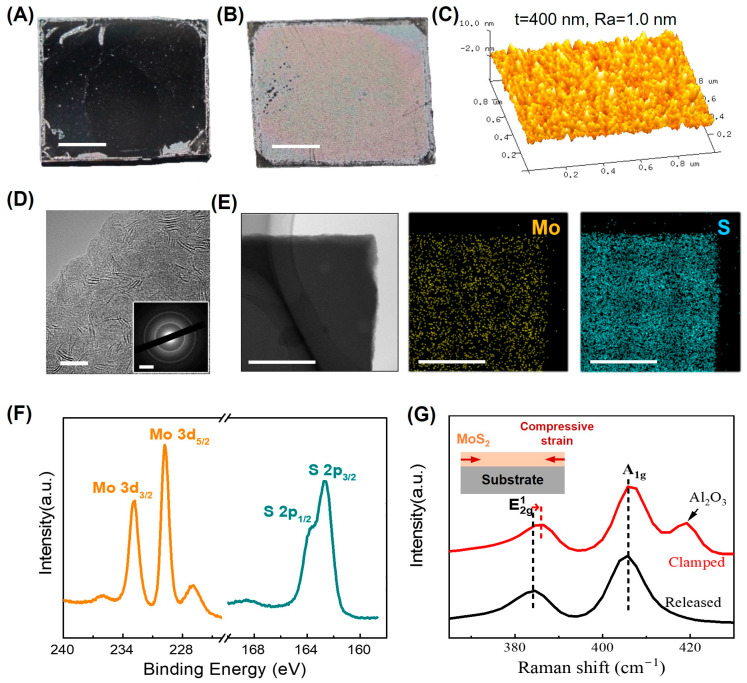
MoS_2_ TFs prepared using PAD. (**A**,**B**) Typical MoS_2_ TFs before and after buckling, indicating a mirror and diffusive reflection, respectively. Scale bars, 3 mm. (**C**) AFM 3D topography of a MoS_2_ thin film grown at 850 °C. (**D**) High-resolution TEM image of the MoS_2_ TFs. The inset shows the selected area electron diffraction (SAED) pattern. Scale bars, 10 nm (**D**); 5.0 nm^−1^ (inset of (**D**)). (**E**) TEM image of MoS_2_ TFs and the corresponding elemental mapping of Mo and S using an energy-dispersive spectrometer (EDS), respectively. Scale bars, 500 nm. (**F**) XPS spectra of Mo 3d and S 2p peaks for the MoS_2_ TFs grown at 850 °C. (**F**,**G**) Raman spectra of as-grown and as-released MoS_2_ TFs (reprinted with permission from [5], Copyright, American Chemical Society).

As early as 2001, Remskar et al. [160] used C_60_ as a growth promoter to prepare chiral MoS_2_ nanotubes using the catalytic transport method. Inspired by this experimental method, Jagličić et al. [161] and Mihailovic et al. [162] each studied the magnetism of Li-doped MoS_2_ nanotube structures in 2003. Li-doped MoS_2_ nanotubes seem to be the realization of a near ideal 1D system, showing a strong new qualitative correlation in behavior. Moreover, edge-oriented MoS_2_ TFs [163,164] exhibit weak magnetism (~1–2 emu∙g*^−^*^1^). The magnetism is related to the edge spin on the edge of the prism of the nanosheet. Remarkably, the first preparation of MoS_2_ TFs [26] using PAD was achieved in 2016. The absorption spectrum showed that MoS_2_ TFs have thickness-dependent band gap regulation. When the film thickness was reduced to 2.5 nm, discontinuities appeared. Inspired by this work, we prepared MoS_2_ TFs [3,5] with adjustable thicknesses, shown in Figure 9. 

Due to the mismatch of thermal expansion coefficients between the film and the substrate, residual compressive strain is introduced at the interface between film and substrate during high temperature growth. After buckling, MoS_2_ TFs change from very flat to rough, as seen in Figure 9A,B. As shown in Figure 9C, the roughness of the 400 nm thick film is about 1 nm. The crystallinity of the film in Figure 9D, in which there are polycrystalline and amorphous components, is not high. However, the distribution of Mo and S elements is relatively uniform (Figure 9E), and their valences are +4 and −2 (Figure 9F), respectively. Raman measurements show that there is compressive strain at the bottom of the film, as seen in Figure 9G.

**Figure 10:**
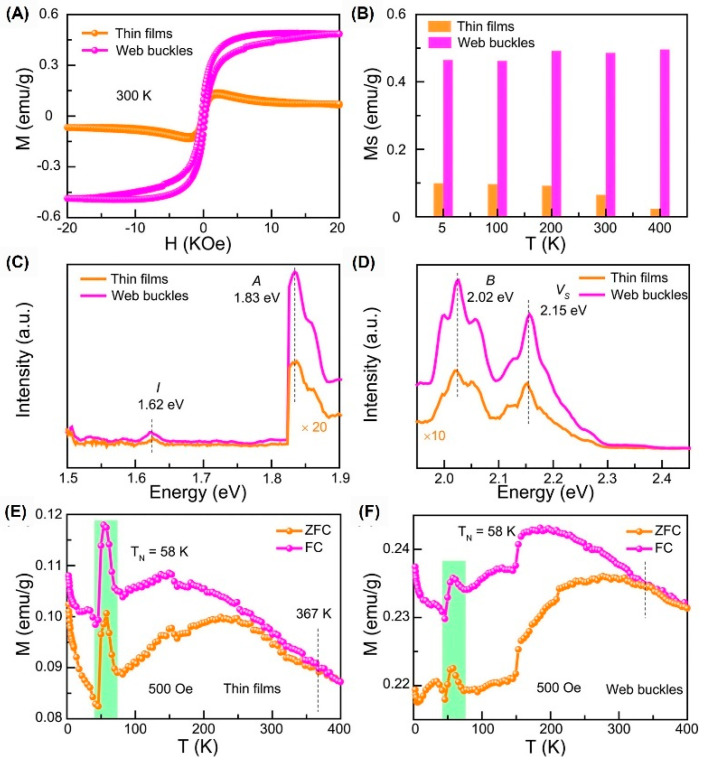
MoS_2_ TFs with RTFM. *M*−*H* curves (**A**), *M_s_*−*T* (**B**), FL excitation spectra (**C**), and FL emission spectra (**D**) of MoS_2_ TFs and WBs. (**E**,**F**) *M*−*T* of MoS_2_ TFs and WBs (reprinted with permission from [5], Copyright, American Institute of Physics).

Due to the disturbance of humidity or temperature, the residual compressive strain in the film is released, resulting in the formation of web buckles [3,4,5,22]. According to the change rate of peak frequency (1/*ꞷ*) × (d*ꞷ*/d*ɛ*), we can estimate the strain change on the surface of the film [3,165]. The film changes from compressive strain to tensile strain after buckling. Interestingly, the RTFM of web buckles is 7.5 times stronger than that of the flat TFs in Figure 10A. In detail, *M_s_* is increased at different test temperatures (Figure 10B). The fluorescence spectra in Figure 10C,D show that the enhanced magnetism is related to *Vs*. Biaxial strain can regulate the magnetic ordering temperature as shown by the dot line, which decreases from 367 K to 338 K after buckling (Figure 10E,F). There exist small protuberances around 57 K in the green area corresponding to the Neel point (*T_N_*), which is the consequence of the antiferromagnetic forces. In general, the decrease in compressive strain and the increase in tensile strain produce more defects, thereby enhancing their RTFM.

### 4.2. MoSe_2_

Recently, MoSe_2_ has received more attention because it has a similar hexagonal atomic-layers structure to MoS_2_ and even better electronic and optical properties because of the more suitable bandgap (~1.1–1.6 eV) [166] compared to MoS_2_ (~1.3–1.9 eV) [167]. Notably, Zhang et al. [168] found a spin-splitting of ~180 meV at the top of the valence of MoSe_2_ film prepared using MBE, which was larger than that of ~100 meV for single-layer MoS_2_ prepared using mechanical exfoliation [169], suggesting MoSe_2_ has a greater application potential than MoS_2_ in spintronic devices. To investigate the magnetism of MoSe_2_, in 2015, Xia et al. [170] synthesized 2H-MoSe_2_ nanoflakes with zigzag edges using CVD, which exhibited *M_s_* of 1.39 emu∙g^−1^ that diminished when the flakes became bigger. Furthermore, they also synthesized 1T-MoSe_2_ incorporated nanosheets [171] and nanoflowers [172] using hydrothermal and solvothermal, respectively, but their *M_s_* values (8.36 × 10^−3^ emu∙g^−1^ and 2.70 × 10^−2^ emu∙g^−1^) were even lower than those in 2H-MoSe_2_ nanoflakes with zigzag edges, despite predictions that 1T-MoSe_2_ provided a magnetic moment of 2 μ_B_/Mo atom equal to that provided by Mo atoms at the zigzag edges [25].

**Figure 11:**
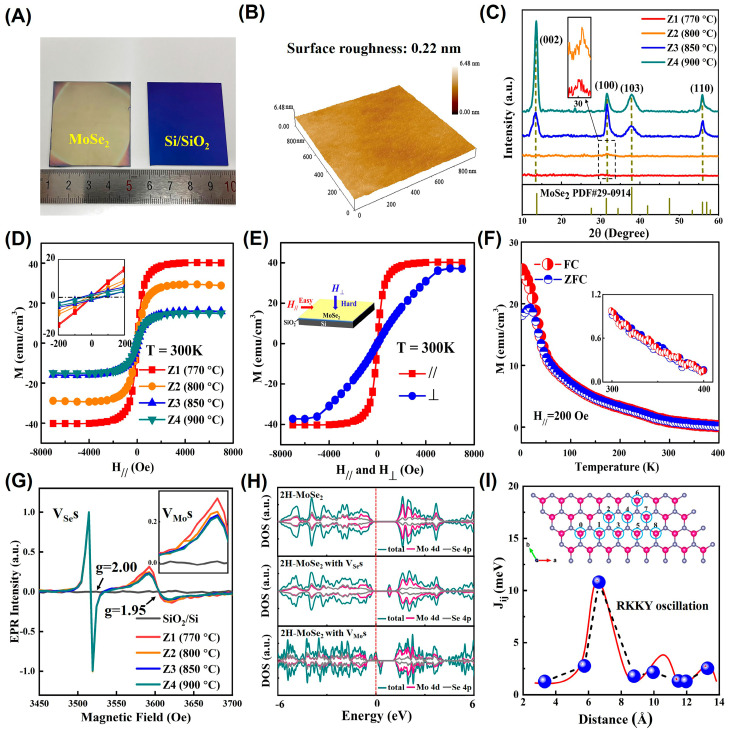
(**A**) Photographs of MoSe_2_ TF and Si/SiO_2_ substrate. (**B**) 3D-AFM image. (**C**) XRD patterns of MoSe_2_ TFs. (**D**) The *M*−*H* curves of MoSe_2_ TFs with *H_//_* applied at 300 K. (**E**) The *M*−*H* curves of MoSe_2_ TF with *H_//_* and *H_⊥_* applied at 300 K. (**F**) The *M*−*T* curves of MoSe_2_ TF under ZFC and FC. (**G**) EPR spectra of MoSe_2_ TFs. (**H**) TDOSs and PDOSs of 2H−MoSe_2_, 2H−MoSe_2_ with V_Se_s, and 2H−MoSe_2_ with V_Mo_s. (**I**) Relationship between *J_ij_* and the *V_Mo_*–*V_Mo_* distance. The inset presents the 8 × 4 × 1 of MoSe_2_ used to calculate *J_ij_*, and the numbers are the thinkable sites of V_Mo_s (reprinted with permission from [25]. Copyright, The Royal Society of Chemistry).

The PAD was first used to synthesize MoSe_2_ TFs in 2020 [173]. A photodetector was fabricated based on the 22 nm thickness of MoSe_2_ TFs on Au substrate, which showed near-perfect absorption in visible wavelengths. Based on the above work, we prepared 4 cm × 4 cm of MoSe_2_ TFs with a smooth surface (roughness average ~0.22 nm) using PAD [25], as shown in Figure 11A,B. 

The crystallinity of the films increases but the *M_s_* decreases with the growth temperature, as shown in Figure 11C,D. Remarkably, amorphous MoSe_2_ TF grown at 770 °C exhibits the largest *M_s_* value of 6.69 emu∙g^−1^, which is about 5 times that of 2H-MoSe_2_ nanoflakes with abundant zigzag edges (1.39 emu∙g^−1^) [170], much higher than that of 1T@2H-MoSe_2_ nanosheets (8.36 × 10^−3^ emu∙g^−1^) [171] and amorphous 1T@2H-MoSe_2_ nanoflowers (2.70 × 10^−2^ emu∙g^−1^) [172]. The *M*−*H* curves measured with *H_//_* and *H_⊥_* applied (Figure 11E) indicate that the magnetic easy axis of MoSe_2_ TFs is in-plane. The field-cooling (FC) and zero-field-cooling (ZFC) *M*−*T* curves of MoSe_2_ TFs (*H_//_* = 200 Oe) shown in Figure 11F indicate that the *T_c_* of MoSe_2_ TFs is higher than 400 K. Figure 11G shows the electron paramagnetic resonance (EPR) spectra of the samples, definitely indicating that *V_Se_*s and *V_Mo_*s are both present in samples and suggesting that there is a relationship between the strong RTFM and the *V_Se_*s and *V_Mo_*s in the MoSe_2_ TFs prepared using PAD. The first-principles calculations are used to further understand the relationship between the FM and the vacancy defects. The spin-resolved total density of states (TDOSs) and partial density of states (PDOSs) of Mo 4d electrons and Se 4p electrons, as shown in Figure 11H, suggest that only 2H-MoSe_2_ with *V_Mo_*s can induce robust magnetism. Figure 11I shows that the relationship between *J_ij_* and *V_Mo_*–*V_Mo_* distances (3.32–13.27 Å) is an RKKY oscillation and the magnetic coupling is always FM for the two-*V_Mo_*s system. To sum up, our experimental and theoretical results both show that the strong RTFM of MoSe_2_ TFs prepared using PAD mainly originates from the RKKY interactions between the magnetic moments of *V_Mo_*s.

### 4.3. ReS_2_

**Figure 12:**
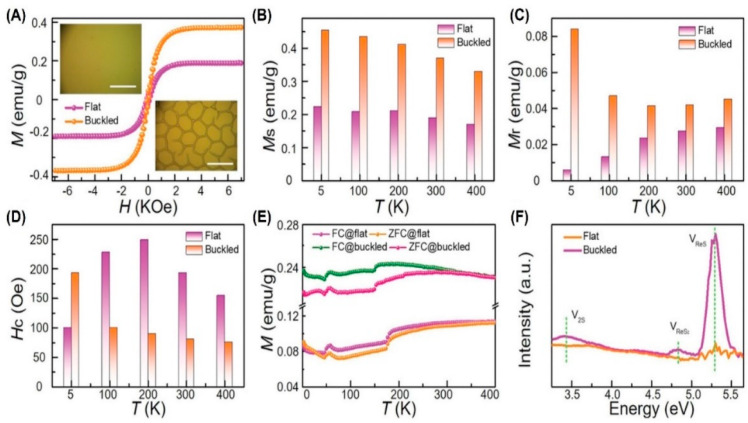
ReS_2_ TFs with RTFM. (**A**) M–H curves at 300 K. Scale bar: 50 µm. (**B**–**D**) *M_s_*−T, *M_r_*−T, and *H_c_*−T, respectively. (**E**) FC and ZFC curves. (**F**) FL curves. Reprinted with permission from [4], Copyright, John Wiley and Sons.

Bulk rhenium disulfide (ReS_2_) is a direct band gap semiconductor [174,175,176] which is pristine and non-magnetic [177,178]. Early research mainly focused on theoretical research, trying to introduce magnetism through defect engineering [177], doping engineering [178,179], and strain engineering. Similar to MoS_2_ web buckles with RTFM, we also prepared large-area ReS_2_ [4] web buckles in Figure 10. The partial release of compressive strain causes the magnetism to increase to 1.69 times, as shown in Figure 12A. After buckling, *M_s_* and *M_r_* are increased in Figure 12B,C. However, *H_c_* is increased only at 5 K and is decreased at other temperatures in Figure 12D. In addition, the in-plane FM of ReS_2_ is weaker than the out-of-plane FM, which is consistent with the ferromagnetic characteristics of 2D materials. The FC and ZFC curves in Figure 12E show that *T_c_* is higher than 400 K. The introduction of biaxial tensile strain is accompanied by more defects, which enhances RTFM in Figure 12F.

## 5. Atomically Thin Non-Layered Materials

### 5.1. NiO

Although 2D zinc oxide [21,29,43,44,71,93] was not successfully grown using PAD, through unremitting efforts, Zou et al. [2] obtained wafer-scale two-dimensional NiO TFs with RTFM in 2021. NiO is an intrinsic p-type semiconductor [180,181], and its bulk is an antiferromagnetism (AFM) [181,182] material. However, the nickel oxide with cation deficiency (Ni_1−x_O) [173,183] exhibits FM. Moreover, local deficient Ni at dislocations [184] could also cause FM in antiferromagnetic NiO single-crystal TFs fabricated using PLD. Similarly, NiO crystalline nanoclusters may be associated with RTFM in sputtered TFs [185]. Actually, previous studies have shown that when the size of NiO nanoparticles (NPs) is reduced to nanoscale, the surface of NPs will induce weak FM [184,186,187]. Interestingly, it was also found that *M_s_* is proportional to the reciprocal of the size of the nanoparticles [188]. 

However, the previously prepared NiO NPs [173,182,183,185,186,187,188,189,190] or TFs [184,185] usually exhibit weak FM, which limits their application in the field of spintronics. Wafer-scale NiO TFs with RTFM are essential for the construction of spintronic devices. The successful preparation of wafer-scale TFs [3,4,5,16,17,19,20,21,22,191,192] is the traditional advantage of PAD. By controlling the concentration of Ni-precursors and the spin-coating rate, sub-nanometer NiO TFs [2] were successfully obtained. When the precursor concentration of Ni was 4.396 mg∙ml^−1^ and the spin-coating rate was 8000 rpm, the thickness of the film was about 0.92 nm. The strong FM hysteresis loops could be observed using SQUID. Moreover, *M_s_* (23.5 emu∙g^−1^ at RT) was almost proportional to the reciprocal of the thickness of TFs, similar to NPs [188]. The lattice defects on the surface of TFs may cause this abnormal magnetic behavior. As a matter of fact, such films with sub-nanometer thickness are polycrystalline.

#### Thickness-Dependent RTFM

**Figure 13:**
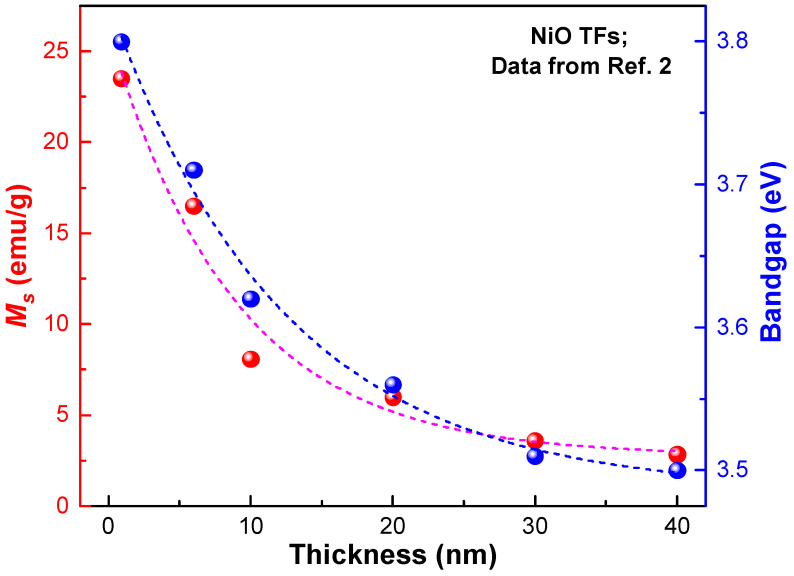
*M_s_* and bandgap of NiO TFs [2] with different thicknesses.

The controllable thickness of thin films is an important feature of PAD. In detail, the thickness can be controlled by the concentration of precursors [2,26] and the spin-coating rate [2,5]. As shown in Figure 13, *M_s_* and bandgap are closely related to the thickness. Furthermore, the total magnetic moment of TFs with different thicknesses seems to be a constant. Interestingly, the thickness can even be adjusted to sub-nanometer level.

## 6. Origin of Ferromagnetism in PAD-Grown Materials

Notably, the FM of PAD-grown materials can be mediated with strain engineering [3,4,22,27,63,67,68], defect engineering [2,21,25,29,43,44,71,78], doping engineering [29,30,97,98], and phase engineering [37], as listed in Table 1. The *T_c_* of most materials could reach room temperature, and the origin of their FM was mainly due to cation defects such as *V_Zn_* [21,29,43,44,72], *V_Mn_* [97,98], *V_Mo_* [3], and *V_Re_* [4].

## 7. Outlook

**Figure 14:**
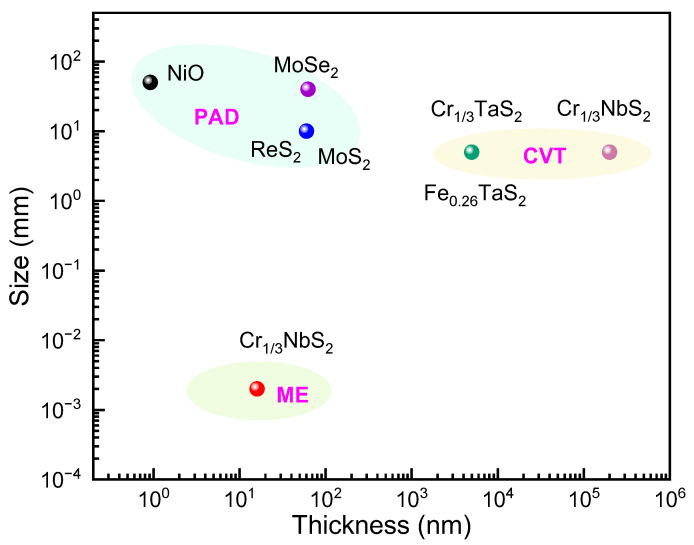
The advantages of PAD in the preparation of chiral magnetic soliton materials. Data from [2,3,4,5,25,26,72,73,74,75].

Take the chiral magnetic soliton CrNbS_1/3_ (CNS) as an example, which is usually prepared using CVT [73,74,75,156] or ME [71]. Although single-crystal materials can be produced using CVT, its growth time is too long; it often takes several days or even a week. Furthermore, it is difficult to produce centimeter-scale materials. In addition, it is difficult to obtain large-size and controllable thickness samples using ME. So far, the thinnest CNS sample obtained was only 16 nm. Obtaining thinner samples is helpful to reveal the mystery of the disappearance of chiral magnetic solitons. However, PAD can prepare TFs with adjustable thickness, which provides the possibility of completing this task, as seen in Figure 14. 

In this review, we summarized the recent developments of 2D ferromagnetic materials fabricated using PAD. Then, we introduced thickness-dependent, strain-modulation, doping, and morphology-dependent RTFM, respectively. Finally, we look forward to the advantages of PAD in the field of chiral magnetic soliton materials. This review provides a novel technique for preparing chiral magnetic soliton materials and may inspire new applications.

## Figures and Tables

**Table 1:** Summary of PAD progress for the synthesis of magnetic materials. Photoluminescence (PL); Vienna ab initio simulations package (VASP); magnetoresistance (MR); ferromagnetic resonance (FMR).

Materials	Magnetic Order	*T_c_*	Origin	Characterization Technique	Strategy
ZnO [71]	FM	>300 K	*V_Zn_*	SQUID; XPS; PAS	Defect engineering
Zn_0.97_Co_0.03_O [29]	FM	>300 K	*V_Zn_*	SQUID; XPS; PL	Defect and doping engineering
ZnO HZNW [43]	FM	>300 K	*V_Zn_*	SQUID	Defect engineering
ZnO VZPA [44]	FM	>300 K	*V_Zn_*	SQUID; PL	Defect engineering
Mn-doped Ga_2_O_3_ [97,98]	FM	~350 K	*Vo*; *Mn*^2*+*^, *Mn*^3*+*^, *Mn*^4*+*^	SQUID; XPS; PL	Defect and doping engineering
SrRuO_3_ [33]	FM	~160 K	unclear	SQUID	Defect engineering
LaCoO_3_ [67]	FM	~85 K	high-spin *Co*^3*+*^	SQUID; MR	Defect and strain engineering
LaCoO_3_ [27]	FM	~85 K	high-spin *Co*^3*+*^	SQUID	Strain engineering
LCMO: NiO [37]	FM	~158 K	lattice parameter	SQUID; MR	Phase engineering
LCMO: Co_3_O_4_ [37]	FM	~210 K	lattice parameter	SQUID; MR	Phase engineering
R_2_CoMnO_6_ [68]	FM	~183 K	chemical and biaxial compressive strain	SQUID	Strain engineering
R_2_NiMnO_6_ [77]	FM	~270 K	cation disorder	SQUID	Strain engineering
Y_3_Fe_5_O_12_ [77]	FM	~500 K	oxygen content	SQUID; FMR	Defect engineering
MoS_2_ [3]	FM	>400 K	*V_Mo_*	SQUID; PL	Defect and strain engineering
MoSe_2_ [25]	FM	>400 K	*V_Mo_*	SQUID; VASP; EPR	Defect engineering
ReS_2_ [4]	FM	>400 K	*V_Re_*, *V_ReS_*, *V_ReS_*_2_	SQUID; VASP	Defect and strain engineering
NiO [2]	FM	~380 K	lattice defects	PPMS; MR	Defect engineering

## Data Availability

Not applicable.
